# Measurement of prostate-specific antigen in detection of benign or malignant breast disease in women

**DOI:** 10.1038/sj.bjc.6690252

**Published:** 1999-03

**Authors:** J Romppanen, R Keskikuru, V Kataja, M Eskelinen, V-M Kosma, K Savolainen, M Uusitupa, I Mononen

**Affiliations:** Department of Clinical Chemistry, Kuopio University Hospital, PO Box 1777, FIN-70211, Finland; Department of Oncology, Kuopio University Hospital, PO Box 1777, FIN-70211, Finland; Department of Surgery, Kuopio University Hospital, PO Box 1777, FIN-70211, Finland; Department of Pathology, Kuopio University Hospital, PO Box 1777, FIN-70211, Finland; Department of Clinical Nutrition, Kuopio University Hospital, PO Box 1777, FIN-70211, Finland

**Keywords:** breast neoplasms, immunoassay, fibroadenoma, mastopathy

## Abstract

Using a highly sensitive chemiluminescent enzyme immunoassay, we have evaluated the measurement of serum prostate-specific antigen (PSA) as a potential diagnostic test for differentiation between women with breast cancer and those with benign breast disease. In a controlled study consisting of 284 women with well-documented patient files and matched for age and long-term place of residence, serum samples collected from 90 women with histologically confirmed breast cancer, 94 women with benign breast disease and 100 controls were analysed. Serum total PSA levels in benign breast disease and cancer patients are not statistically different from those of healthy controls. Total PSA levels decrease with age in normal controls and breast cancer patients but not in those with benign breast disease. The total PSA concentration decreases after menopause in healthy women, though not in patients with breast cancer or benign breast disease. Total PSA bore no relation to the histological type or grade of the tumour or the disease stage of the breast cancer patients. In benign breast disease, all mastopathy patients had normal total PSA, whereas elevation of the values was observed in 7% of fibroadenoma patients. Our results show that serum total PSA cannot be used to distinguish between healthy women and/or women with breast cancer or benign breast disease. © 1999 Cancer Research Campaign


*British Journal of Cancer* (1999) **79**(9/10), 1583–1587
© 1999 Cancer Research Campaign
Article no. bjoc.1998.0252

### Measurement of prostate-specific antigen in detection of benign or malignant breast disease in women

**J Romppanen[1], R Keskikuru[2], V Kataja[2], M Eskelinen[3], V-M Kosma[4], K Savolainen[1], M Uusitupa[5] and I Mononen[1]**

[1]Department of Clinical Chemistry, [2]Department of Oncology, [3]Department of Surgery, [4]Department of Pathology, [5]Department of Clinical Nutrition, Kuopio University Hospital, PO Box 1777, FIN-70211, Finland

**Summary** Using a highly sensitive chemiluminescent enzyme immunoassay, we have evaluated the measurement of serum prostate-specific antigen (PSA) as a potential diagnostic test for differentiation between women with breast cancer and those with benign breast disease. In a controlled study consisting of 284 women with well-documented patient files and matched for age and long-term place of residence, serum samples collected from 90 women with histologically confirmed breast cancer, 94 women with benign breast disease and 100 controls were analysed. Serum total PSA levels in benign breast disease and cancer patients are not statistically different from those of healthy controls. Total PSA levels decrease with age in normal controls and breast cancer patients but not in those with benign breast disease. The total PSA concentration decreases after menopause in healthy women, though not in patients with breast cancer or benign breast disease. Total PSA bore no relation to the histological type or grade of the tumour or the disease stage of the breast cancer patients. In benign breast disease, all mastopathy patients had normal total PSA, whereas elevation of the values was observed in 7% of fibroadenoma patients. Our results show that serum total PSA cannot be used to distinguish between healthy women and/or women with breast cancer or benign breast disease.

**Keywords:** breast neoplasms; immunoassay; fibroadenoma; mastopathy

Prostate-specific antigen (PSA) is a glycoprotein produced by the prostate gland to liquify semen, and low concentrations are normally detected in male serum. Measurement of PSA in serum is widely used as a tumour marker in the diagnosis and monitoring of prostate cancer. Immunoreactive prostate-specific antigen (IR-PSA) has also been detected in other tissues including normal breast, breast cancer (BC) and benign breast disease (BBD) using immunofluorometric (Diamandis et al, 1994; Yu et al, 1995*a*; Yu et al, 1996) and immunohistochemical techniques (Howarth et al, 1997). A high IR-PSA content has been associated with early disease stage (Yu et al, 1996), small tumours (Yu et al, 1995*a*; Yu et al, 1998) and oestrogen and/or progesterone receptor-positive breast tumours (Diamandis et al, 1994). Major expression of PSA has also been detected in BBD tissue (Yu et al, 1996). IR-PSA-positivity has been suggested as a favourable prognostic indicator for women with BC (Yu et al, 1995*a*; Yu et al, 1998) and a marker of steroid hormone action in normal and diseased female breast (Yu et al, 1996).

Sera from healthy women contain small amounts of PSA that is mostly complexed with $\alpha_1$-antichymotrypsin (PSA-ACT) (Diamandis et al, 1996; Melegos and Diamandis, 1996). About half of the patients with BC and BBD have been reported to have free PSA as the major molecular form in their serum (Borchert et al, 1997*a*; Melegos and Diamandis, 1996). Giai et al (1995) have reported that serum PSA in women with BC was not associated with tumour PSA levels and that for women ≥50 years, there was no difference in serum PSA between normal and BC patients. Recently, Borchert et al (1997*b*) reported that serum total PSA levels in patients with ductal carcinoma in situ are higher than in patients with ductal carcinoma or other types of breast carcinoma. It is also higher in serum of patients with poorly differentiated carcinomas (grade 3) compared with grade 1 or 2 tumours (Borchert et al, 1997*b*). Women with BBD seemed to have significantly higher serum total PSA levels than female blood donors or cancer patients (Borchert et al, 1997*b*).

Exceptionally high serum PSA levels have been observed in subjects with fibroadenomas and breast cysts (Borchert et al, 1997*a*). These findings suggest that high serum levels of PSA in women might be a valuable diagnostic and prognostic marker for BC, fibroadenomas and breast cysts (Borchert et al, 1997*a*; Melegos and Diamandis, 1996; Yu et al, 1995*a*). In this study we have evaluated the measurement of serum PSA as a potential test for differentiating between healthy women and those with BC or BBD.

### MATERIALS AND METHODS

### Serum samples

The Kuopio Breast Cancer Study is a prospective, still ongoing case–control study with the participation of 2500 women. From these subjects, 90 women with histologically confirmed BC without any other malignancy, 94 women with BBD and 100 healthy women (population controls) were randomly selected for the present study. Table 1 shows the clinical data that were collected from the patient files. Patients with BBD consisted of 47 patients with mastopathy (including 38 women with fibrocystic disease, 6 women with lipoma, 2 women with periductal mastitis

*Received 18 May 1998*
*Revised 7 September 1998*
*Accepted 29 September 1998*

*Correspondence to:* I Mononen

**Table 1** Characteristics of the healthy population control (Co), benign breast disease (BBD) and breast cancer (BC) groups

| Panel A | Co | BBD | BC |
|---|---|---|---|
| Number | 100 | 94 | 90 |
| Mean age (range) | 55.2 (32–77) | 41.8 (16–75) | 59.6 (31–89) |
| Post-menopausal, number (%) | 59 (59.0) | 22 (23.4) | 62 (68.9) |
| ERT[a], number (%) | 18 (18.0) | 17 (18.1) | 19 (21.1) |
| BBD diagnosis, number (%) | | | |
| Mastopathy (M) | | 47 (50.0) | |
| Fibroadenoma (F) | | 44 (46.8) | |
| Other (O) | | 3 (3.2) | |
| BC diagnosis, number (%) | | | |
| Ductal carcinoma (DC) | | | 56 (62.2) |
| Lobular carcinoma (LC) | | | 13 (14.4) |
| Ductal carcinoma in situ (DCIS) | | | 15 (16.7) |
| Lobular carcinoma in situ (LCIS) | | | 2 (2.2) |
| Other (O) | | | 4 (4.4) |
| BC stage, number (%) | | | |
| Ca in situ | | | 17 (18.9) |
| I | | | 18 (20.0) |
| II | | | 19 (21.1) |
| III | | | 24 (26.7) |
| IV | | | 12 (13.3) |
| Invasive BC histological grade, number (%) | | | |
| I | | | 14 (19.2) |
| II | | | 38 (52.1) |
| III | | | 19 (26.0) |
| Not available | | | 2 (2.7) |

[a]ERT = oestrogen replacement therapy.

and 1 woman with fat necrosis), 44 patients with fibroadenoma, and two patients with adenoma and one patient with papillomatosis. The population controls were matched with the BC patients for age (± 5 years) and long-term place of residence (rural vs. urban). All the blood samples were collected by venipuncture and, after separation, the sera were stored frozen (– 20°C) until analysed. The serum samples from patients with BC or BBD were obtained prior to any surgical or other therapeutic procedures. All the procedures were in accordance with the Helsinki Declaration of 1975, as revised in 1983, and all the study subjects had signed an informed consent form.

### PSA immunoassay

The total PSA concentrations in sera were measured with an IMMULITE Third Generation PSA chemiluminescent immunoassay system (Diagnostic Products Corporation, Los Angeles, CA, cat. no. LKUP1). The lower detection limit of the total PSA assay, defined as the concentration two standard deviations above the response at zero dose, is 3 ng $l^{-1}$ and the functional sensitivity (coefficient of variation less than 20%) was 5 ng $l^{-1}$ (DPC Immulite Third Generation PSA product profile). The PSA method does not crossreact with AFP, CEA, ferritin, hCG, lactalbumin, prostatic acid phosphatase or prolactin (DPC Immulite Third Generation PSA product profile).

### Statistics

The Kruskal–Wallis one-way variance analysis was used for median comparisons of total PSA values between patient groups. The differences between groups in contingency tables were tested by Fisher exact test. Effect of menopause and age on PSA values were tested with Spearman correlation analysis. The Pearson correlation analysis was used to test the effect of age on log (total PSA) values.

### RESULTS

### Prostate-specific antigen in serum of breast cancer and benign breast disease patients

Table 2 shows the distribution of total PSA values in serum of the healthy controls and patients with BBD or BC. No statistically significant differences were found between the groups ($P > 0.05$). Four per cent of the healthy controls, 3.2% of the BBD patients and 5.6% of the BC patients had total PSA values over 30 ng $l^{-1}$; but these differences were not statistically significant ($P > 0.05$).

**Table 2** Distribution of total PSA in controls (Co), benign breast disease (BBD) and breast cancer (BC) patients

| | | Percentile of total PSA (ng $l^{-1}$) | | | | | | |
|---|---|---|---|---|---|---|---|---|
| Patient group | *N* | 0 | 5 | 25 | 50 | 75 | 95 | 100 |
| Co | 100 | 3.0 | 3.0 | 3.0 | 4.0 | 7.0 | 24.0 | 83.0 |
| BBD | 94 | 3.0 | 3.0 | 4.0 | 5.0 | 10.0 | 21.0 | > 20 000 |
| BC | 90 | 3.0 | 3.0 | 3.0 | 5.0 | 8.0 | 40.0 | 422.0 |

*© Cancer Research Campaign 1999*

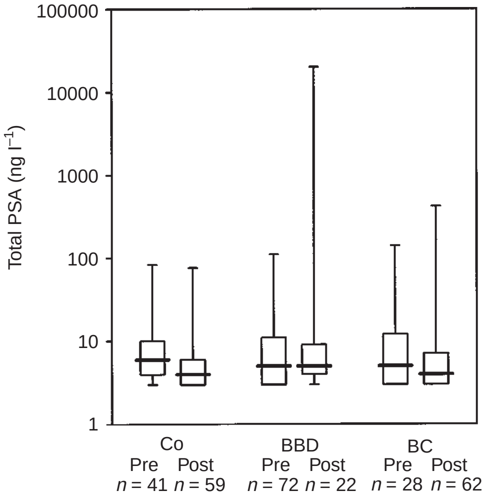

**Figure 1** Concentration of total PSA in serum of pre- (Pre) and post-menopausal (Post) healthy women (Co; $n$ = 100), female patients with BBD ($n$ = 94) and women with BC ($n$ = 90). Boxplots show the median, interquartile range, and minimum and maximum values in each group. Total PSA level in the control group was significantly higher in premenopausal women than in post-menopausal subjects ($P < 0.01$)

### Effect of menopause and age on PSA values

Total serum PSA level in pre- and post-menopausal women is shown in Figure 1. In the control group, but not in the BBD or BC patients, the PSA level was significantly higher in premenopausal women than in post-menopausal counterparts ($P < 0.01$). A significant negative correlation between total PSA and increasing age was found in the control group (Spearman's rho = – 0.31; $P < 0.005$) and BC patient group (Spearman's rho = – 0.42; $P < 0.001$), but not in the BBD group (Spearman's rho = – 0.016; $P > 0.05$). The same phenomenon was observed between log (total PSA) and increasing age in the control group (Pearson's correlation coefficient = – 0.23; $P < 0.05$) and BC patient group (Pearson's correlation coefficient = – 0.32; $P < 0.005$), but not in the BBD group (Pearson's correlation coefficient = 0.00; $P > 0.05$).

### Correlation of clinical data with serum PSA values

Table 3 shows the clinical data on those three BBD patients, six BC patients and five controls who had serum total PSA values > 24 ng $l^{-1}$. High total PSA bore no relationship to the clinical status of the BC patients (Table 3).

Patients with ductal carcinoma tend to have serum total PSA levels higher than patients with ductal carcinoma in situ (Figure 2A), but the difference was not statistically significant. There was not any significant correlation or differences between total PSA values and histological grade (Figure 2B) or disease stage (Figure 2C) of BC.

In BBD, all the 47 patients with mastopathy had their serum total PSA concentration (median = 5 ng $l^{-1}$; the interquartile range = 4–9 ng $l^{-1}$; 95th percentile = 16 ng $l^{-1}$) within the 95th percentile of the values of healthy women. The median of total PSA values in the patients with fibroadenomas ($n$ = 44) was also 5 ng $l^{-1}$ (the interquartile range 3–11 ng $l^{-1}$), but in 3/44 patients the serum total PSA concentration was above the 95th percentile of the values of healthy controls (24 ng $l^{-1}$) ranging from 80 ng $l^{-1}$ to more than 20 000 ng $l^{-1}$ (Table 3).

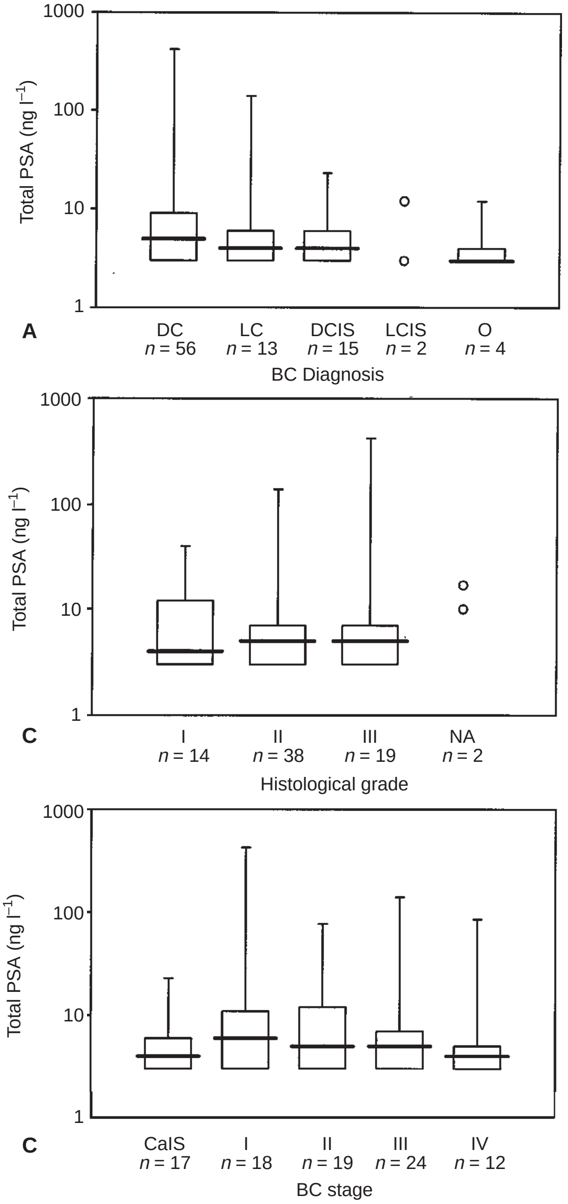

**Figure 2** Total PSA levels in the serum of BC patients with different histological types (**A**), histological grades (**B**), and stages of BC (**C**). Patients with ductal carcinoma tend to have serum total PSA levels higher than those with ductal carcinoma in situ, although the difference was not significant ($P > 0.05$). Total PSA levels did not reveal any significant differences between different histological grades or different stages of BC. Abbreviations as in Table 1; NA = not analysed

### DISCUSSION

Earlier reports have suggested that IR-PSA-positivity of breast tumours is a favourable prognostic indicator for women with BC (Giai et al, 1995; Yu et al, 1995*a*, 1998) and that high serum levels of PSA in women might represent a valuable diagnostic marker in BC (Borchert et al, 1997*b*; Melegos and Diamandis, 1996) as well

© Cancer Research Campaign 1999

**Table 3** Characteristics of the individual control persons (Co) and benign breast disease (BBD) and breast cancer (BC) patients with total PSA > 24 ng $l^{-1}$ in serum

| | Co 1 | Co 2 | Co 3 | Co 4 | Co 5 | BBD 1 | BBD 2 | BBD 3 | BC 1 | BC 2 | BC 3 | BC 4 | BC 5 | BC 6 |
|---|---|---|---|---|---|---|---|---|---|---|---|---|---|---|
| Diagnosis[a] | | | | | | (F) | (F) | (F) | (DC) | (DC) | (LC) | (DC) | (DC) | (DC) |
| Total PSA[b] | 50 | 47 | 76 | 30 | 83 | > 20 000 | 110 | 80 | 422 | 85 | 140 | 77 | 28 | 40 |
| Age | 60.0 | 53.2 | 67.5 | 39.8 | 41.0 | 44.5 | 31.4 | 42.1 | 53.3 | 50.1 | 55.4 | 33.3 | 48.7 | 55.5 |
| Menopausal status | Post | Post | Post | Pre | Pre | Post | Pre | Post | Post | Post | Pre | Pre | Pre | Post |
| ERT[c] | No | No | No | No | No | Yes | No | No | No | No | No | No | No | Yes |
| ER/PR[d] | | | | | | | | | ?/? | –/– | +/– | –/+ | –/+ | ?/? |
| TNM | | | | | | | | | T1N0M0 | T4N2M1 | T3N1M0 | T2N1M0 | T3N0M0 | T1N0M0 |
| Stage | | | | | | | | | I | IV | III | II | III | I |
| Grade | | | | | | | | | III | III | III | II | II | I |

[a]Diagnosis (M), (F), (O), (DC) and (LC) as in Table 1. [b]Total PSA (ng $l^{-1}$). [c]ERT = oestrogen replacement therapy. [d]ER/PR = oestrogen/progesterone receptor status; positive (+), negative (–) or unknown (?).

as fibroadenomas and breast cysts (Borchert et al, 1997*a*; Borchert et al, 1997*b*). However, discordant data concerning the clinical significance of PSA immunoreactivity in breast tumour cytosols (Astill et al, 1996; Dibbelt et al, 1996; Wu et al, 1995) and in breast cysts (Filella et al, 1996; Lai et al, 1996; Mannello et al, 1996) as well as the diagnostic value of serum total PSA in BC (Giai et al, 1995) and breast cyst disease (Filella et al, 1996) have been presented. Most of the earlier results have been obtained using a time-resolved immunofluorometric (TR-FIA) PSA assay (Borchert et al, 1997*b*; Diamandis et al, 1994; Ferguson et al, 1996; Melegos and Diamandis, 1996; Yu et al, 1995*a*, 1996, 1998) which is not commercially available. In this study we have used a commercially available ultrasensitive chemiluminescent enzyme immunoassay system that has been shown to have sensitivity and specificity very close to those of the TR-FIA method (Ferguson et al, 1996; Witherspoon and Lapeyrolerie, 1997) and to detect reliably total PSA in female serum (Borchert et al, 1997*a*; Ferguson et al, 1996; Witherspoon and Lapeyrolerie, 1997). The PSA concentration in female serum is very low and in the range of the lower limit of detection of the currently available PSA assay systems. Any differences that may be attributable to limitations of the sensitivity of the PSA assays remain undetected.

The representative patient and control groups of the present study consisted of 284 women, who were randomly selected from a prospective, still ongoing case–control study with the participation of 2500 women. The clinical data were collected from well-defined patient files of 90 women with histologically confirmed breast cancer (BC) without any other malignancy, 94 women with benign breast disease (BBD) and 100 healthy women (population controls) and the population controls were matched with the BC patients for age and long-term place of residence.

Our results show that a cut-off level of over 30 ng $l^{-1}$ in total PSA values was exceeded in 4.0% of the healthy controls, 3.2% of the BBD patients and 5.6% of the BC, these differences between the groups not being statistically significant. This does not agree with a recent report, in which the percentage of female blood donors (representing normal women) with PSA > 30 ng $l^{-1}$ was significantly lower than the percentage of BC or BBD (Borchert et al, 1997*b*), and with another report, in which the percentage of female blood donors between 17 and 69 years of age (representing normal women) with PSA > 30 ng $l^{-1}$ was significantly lower than the percentage of BC patients aged between 29 and 93 before and post surgery (Giai et al, 1995). The recent observation that serum total PSA levels in patients with ductal carcinoma in situ are higher than in patients with ductal carcinoma or other types of breast carcinoma and in serum of patients with poorly differentiated carcinomas (grade 3) compared with grade 1 or 2 tumours (Borchert et al, 1997*b*) also remained unconfirmed since high total PSA levels bore no relation to the clinical status of BC patients in this present study. Total PSA median in our healthy controls was 4 ng $l^{-1}$, which is slightly higher than the total PSA median (2.0 ng $l^{-1}$) reported in a group of female blood donors in a previous study (Borchert et al, 1997*b*). In our study, the total PSA median in the group of BC patients was 5 ng $l^{-1}$, which is also higher than the median value (1.8 ng $l^{-1}$) reported by Borchert et al. These differences in medians may be due to differences in standardization of the total PSA assays.

Exceptionally high serum PSA levels have been reported in women with fibroadenomas and breast cysts (Borchert et al, 1997*a*). In another study, women with BBD ($n$ = 199) had significantly higher serum total PSA levels than female blood donors ($n$ = 213) or cancer patients ($n$ = 199) (Borchert et al, 1997*b*). In the present study material, one patient with fibroadenoma had PSA levels in the range of males with prostate cancer. The patient was receiving oestrogen replacement therapy, which is interesting in light of reports suggesting that there is hormonal regulation of PSA (Yu et al, 1995*b*; Zarghami et al, 1997). In the present study, elevation of total PSA was detected only in 7% of the fibroadenoma and in none of the mastopathy patients. This shows that elevated serum total PSA levels are not a consistent finding in patients with benign breast disease, which is in accordance with a previous report (Filella et al, 1996). The discrepancies between these results and previous findings (Borchert et al, 1997*a*, 1997*b*) may be due to the selection of the patients, differences in the normal control groups (e.g. population controls in our case–control study vs female blood donors in previous studies) and/or our slightly less sensitive assay system.

Total PSA levels decrease with age in normal controls and breast cancer patients but not in those with benign breast disease. Additionally, total PSA levels were significantly higher in

 © Cancer Research Campaign 1999

premenopausal women than in post-menopausal ones in the control group, but not in the BC and BBD patients. These findings may be further indications that PSA is a marker of steroid hormone action in normal breast (Yu et al, 1995*b*), but the response is weaker in BC and virtually absent in BBD tissue or that the leakage of PSA out of diseased breast tissue may be higher than from normal breast tissue.

The combined evidence demonstrates that a single measurement of the serum concentration of PSA in women detects high PSA values in healthy women and female patients with both benign and malignant breast disease. Thus, the total PSA concentration in serum cannot distinguish between healthy women and/or women with BC or BBD

### ACKNOWLEDGEMENTS

This work was financially supported by a grant from Sigrid Juselius Foundation to IM.

### REFERENCES

Astill ME, Rapp E, Wilson LW, Bryson L and Wu JT (1996) PSA in breast tumour cytosol: a positive prognostic factor? [Abstract]. *Clin Chem* **42**: S266

Borchert GH, Giai M and Diamandis EP (1997*a*) Elevated levels of prostate-specific antigen in serum of women with fibroadenomas and breast cysts. *J Natl Cancer Inst* **89**: 587–588

Borchert GH, Melegos DN, Tomlinson G, Giai M, Roagna R, Ponzone R, Sgro L and Diamandis EP (1997*b*) Molecular forms of prostate-specific antigen in the serum of women with benign and malignant breast diseases. *Br J Cancer* **76**: 1087–1094

Diamandis EP, Yu H and Melegos DN (1996) Ultrasensitive prostate-specific antigen assays and their clinical application. *Clin Chem* **42**: 853–857

Diamandis EP, Yu H and Sutherland DJA (1994) Detection of prostate-specific antigen immunoreactivity in breast tumors. *Breast Cancer Res Treat* **32**: 301–310

Dibbelt L, Vierke G and Wunsche W (1996) Prostate-specific antigen immunoreactivity in women with breast cancer [letter]. *Clin Chem* **42**: 1721–1722

Ferguson RA, Yu H, Kalyvas M, Zammit S and Diamandis EP (1996) Ultrasensitive detection of prostate-specific antigen by a time-resolved immunofluorometric assay and the Immulite immunochemiluminescent third-generation assay: potential applications in prostate and breast cancers. *Clin Chem* **42**: 675–684

Filella X, Molina R, Alcover J, Carretero P and Ballesta AM (1996) Detection of nonprostatic PSA in serum and nonserum samples from women. *Int J Cancer* **68**: 424–427

Giai M, Yu H, Roagna R, Ponzone R, Katsaros D, Levesque MA and Diamandis EP (1995) Prostate-specific antigen in serum of women with breast cancer. *Br J Cancer* **72**: 728–731

Howarth DJC, Aronson IB and Diamandis EP (1997) Immunohistochemical localization of prostate-specific antigen in benign and malignant breast tissues. *Br J Cancer* **75**: 1646–1651

Lai LC, Erbas H, Lennard TW and Peaston RT (1996) Prostate-specific antigen in breast cyst fluid: possible role of prostate-specific antigen in hormone-dependent breast cancer. *Int J Cancer* **66**: 743–746

Mannello F, Bocchiotti G, Bianchi G, Marcheggiani F and Gazzanelli G (1996) Quantification of prostate-specific antigen immunoreactivity in human breast cyst fluids. *Breast Cancer Res Treat* **38**: 247–252

Melegos DN and Diamandis EP (1996) Diagnostic value of molecular forms of prostate-specific antigen for female breast cancer. *Clin Biochem* **29**: 193–200

Witherspoon LR and Lapeyrolerie T (1997) Sensitive prostate specific antigen measurements identify men with long disease-free intervals and differentiate aggressive from indolent cancer recurrences within 2 years after radical prostatectomy. *J Urol* **157**: 1322–1328

Wu JT, Zhang P, Astill ME, Wilson LW, Lyons BW, Wu LL and Stephenson R (1995) PSA immunoreactivity detected in LNCaP cell medium, breast tumor cytosol, and female serum. *J Clin Lab Anal* **9**: 243–251

Yu H, Giai M, Diamandis EP, Katsaros D, Sutherland DJA, Levesque MA, Roagna R, Ponzone R and Sismondi P (1995*a*) Prostate-specific antigen is a new favorable prognostic indicator for women with breast cancer. *Cancer Res* **55**: 2104–2110

Yu H, Diamandis EP, Monne M and Croce CM (1995*b*) Oral contraceptive-induced expression of prostate-specific antigen in the female breast. *J Biol Chem* **270**: 6615–6618

Yu H, Diamandis EP, Levesque M, Giai M, Roagna R, Ponzone R, Sismondi P, Monne M and Croce CM (1996) Prostate specific antigen in breast cancer, benign breast disease and normal breast tissue. *Breast Cancer Res Treat* **40**: 171–178

Yu H, Levesque MA, Clark GM and Diamandis EP (1998) Prognostic value of prostate-specific antigen for women with breast cancer: a large United States cohort study. *Clin Cancer Res* **4**: 1489–1497

Zarghami N, Grass L and Diamandis EP (1997) Steroid hormone regulation of prostate-specific antigen gene expression in breast cancer. *Br J Cancer* **75**: 579–588

*© Cancer Research Campaign 1999*

## References

[PDF_00413] Borchert G. H., Giai M., Diamandis E. P. (1997). Elevated levels of prostate-specific antigen in serum of women with fibroadenomas and breast cysts.. J Natl Cancer Inst.

[PDF_00416] Borchert G. H., Melegos D. N., Tomlinson G., Giai M., Roagna R., Ponzone R., Sgro L., Diamandis E. P. (1997). Molecular forms of prostate-specific antigen in the serum of women with benign and malignant breast diseases.. Br J Cancer.

[PDF_00420] Diamandis E. P., Yu H., Melegos D. N. (1996). Ultrasensitive prostate-specific antigen assays and their clinical application.. Clin Chem.

[PDF_00422] Diamandis E. P., Yu H., Sutherland D. J. (1994). Detection of prostate-specific antigen immunoreactivity in breast tumors.. Breast Cancer Res Treat.

[PDF_00425] Dibbelt L., Vierke G., Wunsche W. (1996). Prostate-specific antigen immunoreactivity in women with breast cancer.. Clin Chem.

[PDF_00428] Ferguson R. A., Yu H., Kalyvas M., Zammit S., Diamandis E. P. (1996). Ultrasensitive detection of prostate-specific antigen by a time-resolved immunofluorometric assay and the Immulite immunochemiluminescent third-generation assay: potential applications in prostate and breast cancers.. Clin Chem.

[PDF_00432] Filella X., Molina R., Alcover J., Carretero P., Ballesta A. M. (1996). Detection of nonprostatic PSA in serum and nonserum samples from women.. Int J Cancer.

[PDF_00435] Giai M., Yu H., Roagna R., Ponzone R., Katsaros D., Levesque M. A., Diamandis E. P. (1995). Prostate-specific antigen in serum of women with breast cancer.. Br J Cancer.

[PDF_00438] Howarth D. J., Aronson I. B., Diamandis E. P. (1997). Immunohistochemical localization of prostate-specific antigen in benign and malignant breast tissues.. Br J Cancer.

[PDF_00441] Lai L. C., Erbas H., Lennard T. W., Peaston R. T. (1996). Prostate-specific antigen in breast cyst fluid: possible role of prostate-specific antigen in hormone-dependent breast cancer.. Int J Cancer.

[PDF_00444] Mannello F., Bocchiotti G., Bianchi G., Marcheggiani F., Gazzanelli G. (1996). Quantification of prostate-specific antigen immunoreactivity in human breast cyst fluids.. Breast Cancer Res Treat.

[PDF_00447] Melegos D. N., Diamandis E. P. (1996). Diagnostic value of molecular forms of prostate-specific antigen for female breast cancer.. Clin Biochem.

[PDF_00449] Witherspoon L. R., Lapeyrolerie T. (1997). Sensitive prostate specific antigen measurements identify men with long disease-free intervals and differentiate aggressive from indolent cancer recurrences within 2 years after radical prostatectomy.. J Urol.

[PDF_00453] Wu J. T., Zhang P., Astill M. E., Wilson L. W., Lyons B. W., Wu L. L., Stephenson R. (1995). PSA immunoreactivity detected in LNCaP cell medium, breast tumor cytosol, and female serum.. J Clin Lab Anal.

[PDF_00463] Yu H., Diamandis E. P., Levesque M., Giai M., Roagna R., Ponzone R., Sismondi P., Monne M., Croce C. M. (1996). Prostate specific antigen in breast cancer, benign breast disease and normal breast tissue.. Breast Cancer Res Treat.

[PDF_00460] Yu H., Diamandis E. P., Monne M., Croce C. M. (1995). Oral contraceptive-induced expression of prostate-specific antigen in the female breast.. J Biol Chem.

[PDF_00456] Yu H., Giai M., Diamandis E. P., Katsaros D., Sutherland D. J., Levesque M. A., Roagna R., Ponzone R., Sismondi P. (1995). Prostate-specific antigen is a new favorable prognostic indicator for women with breast cancer.. Cancer Res.

[PDF_00466] Yu H., Levesque M. A., Clark G. M., Diamandis E. P. (1998). Prognostic value of prostate-specific antigen for women with breast cancer: a large United States cohort study.. Clin Cancer Res.

[PDF_00469] Zarghami N., Grass L., Diamandis E. P. (1997). Steroid hormone regulation of prostate-specific antigen gene expression in breast cancer.. Br J Cancer.

